# The Effects of Nutrient Enrichment and Herbivore Abundance on the Ability of Turf Algae to Overgrow Coral in the Caribbean

**DOI:** 10.1371/journal.pone.0014312

**Published:** 2010-12-13

**Authors:** Mark J. A. Vermeij, Imke van Moorselaar, Sarah Engelhard, Christine Hörnlein, Sophie M. Vonk, Petra M. Visser

**Affiliations:** 1 Carmabi Foundation, Willemstad, Curaçao; 2 Aquatic Microbiology, Institute for Biodiversity and Ecosystem Dynamics, University of Amsterdam, Amsterdam, The Netherlands; University of Glamorgan, United Kingdom

## Abstract

Turf algae are multispecies communities of small marine macrophytes that are becoming a dominant component of coral reef communities around the world. To assess the impact of turf algae on corals, we investigated the effects of increased nutrients (eutrophication) on the interaction between the Caribbean coral *Montastraea annularis* and turf algae at their growth boundary. We also assessed whether herbivores are capable of reducing the abundance of turf algae at coral-algae boundaries. We found that turf algae cause visible (overgrowth) and invisible negative effects (reduced fitness) on neighbouring corals. Corals can overgrow neighbouring turf algae very slowly (at a rate of 0.12 mm 3 wk^−1^) at ambient nutrient concentrations, but turf algae overgrew corals (at a rate of 0.34 mm 3 wk^−1^) when nutrients were experimentally increased. Exclusion of herbivores had no measurable effect on the rate turf algae overgrew corals. We also used PAM fluorometry (a common approach for measuring of a colony's “fitness”) to detect the effects of turf algae on the photophysiology of neighboring corals. Turf algae always reduced the effective photochemical efficiency of neighbouring corals, regardless of nutrient and/or herbivore conditions. The findings that herbivores are not capable of controlling the abundance of turf algae and that nutrient enrichment gives turf algae an overall competitive advantage over corals together have serious implications for the health of Caribbean coral reef systems. At ambient nutrient levels, traditional conservation measures aimed at reversing coral-to-algae phase shifts by reducing algal abundance (i.e., increasing herbivore populations by establishing Marine Protected Areas or tightening fishing regulations) will not necessarily reduce the negative impact of turf algae on local coral communities. Because turf algae have become the most abundant benthic group on Curaçao (and likely elsewhere in the Caribbean), new conservation strategies are required to mitigate their negative impact on coral communities.

## Introduction

As coral reefs degrade, turf algae and macro algae become more abundant and coral cover declines, a phenomenon commonly referred to as a “coral-algal phase shift” (e.g., [1]–[4]). The appearance of algae on substrate previously occupied by corals is often interpreted as evidence that algae actively outcompete corals for space. Alternatively, algae could colonize open space after a coral has already died (e.g., from diseases, storms). Under this scenario, increased algal abundance is a consequence rather than a cause of decreased coral cover [5]. To unequivocally show that competition occurs between corals and algae, one needs experimental proof that algae actively cause the decreased abundance of corals, through e.g., overgrowth, shading, allelopathy. This process must be studied at the spatial and temporal scales on which these interactions occur [6].

For sessile organisms such as terrestrial plants, marine macrophytes and corals, the degree to which species interact depends on their relative abundance and spatial configuration [7]. On reefs, human-induced changes in the abundance of algae and reef building corals (e.g., [1], [2], [4], [8]) have altered the competitive landscape so that corals more often face encroaching algae in their vicinity. Studies of coral-algal phase shifts generally focus on larger macroalgae (e.g., [2], [9], [10]). Smaller turf algae are not always quantified or considered despite the fact that they have become one of the most abundant benthic functional groups on reef communities worldwide [4], [5], [8], [11]–[13] and can be abundant even on near-pristine reefs [14]. Studying the direct effect of turf algae on neighbouring corals will help us understand the extent to which their growth accelerates coral-algal phase shifts.

Turf algae (or “algal turfs”) are dense, multi-species assemblages of filamentous benthic algae and cyanobacteria that are typically less than 1 cm in height [15]. Compared to macro algae, turf algae grow faster [16], occupy newly available space faster [17], [18] and are less vulnerable to physical stress caused by water turbulence [17], [19] and grazing [15], [20]. Furthermore, turf algae prevent successful settlement of newly arriving coral planulae [21], [22] and certain turf algal species can rapidly overgrow and kill coral [23], [24]. Similar effects are less common for macroalgae [23]–[25] and turf algae generally “win” more often in direct interactions with coral relative to macro algae species [14]. Turf algae can weaken and subsequently overgrow neighbouring corals through allelopathic effects, the induction of hypoxia and/or shading [14], [23]–[27].

While algal abundance increases on reefs around the world, the environmental factors driving the outcome of algae-coral competition are not well understood [5], [26], [28]. Overfishing of herbivorous fish and increased eutrophication both increase the abundance of macro algae (e.g., [8], [29], [30]) and these two factors can be expected to influence the abundance of the small macrophytes that comprise turf algae as well [9], [16], [31], [32]; but see: [33], [34]. However, the influence of environmental conditions associated with degrading reefs (e.g., eutrophication and overharvesting of herbivores) on the outcome of competitive interactions between corals and turf algae is presently not well studied (but see: [35], [36]).

Here, we investigated firstly whether Caribbean turf algae negatively affect neighbouring corals either through overgrowth and/or by lowering the coral's fitness, and secondly whether the outcome of such interaction was dependent on the local abundance of herbivorous fish and nutrients. We focused on communities of turf algae (heterogeneous assemblages of filamentous algal and cyanobacterial species on average less than 10 mm in height) [15], [37] that interacted with the dominant Caribbean reef building coral *Montastraea annularis*.

## Materials and Methods

### Ethics statement

This was an observational study conducted in an area that does not require approval from an official body; permits were therefore not required.

### Study location and sites

**Figure 1:**
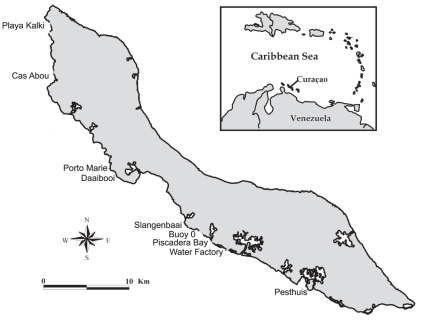
Overview of the surveyed sites along Curaçao's south-west shore. Buoy 0 is the location where the herbivore exclusion experiment was conducted but the benthic community was not surveyed at this site.

Experiments and surveys were carried out between March and May 2009 along the leeward coast of the island Curaçao (12°N 69°W), Netherlands Antilles. An herbivore exclusion experiment was conducted at the site Buoy 0 (Figure 1) and a nutrient enrichment experiment was conducted near the Water Factory, located 2.5 km east of Buoy 0. Nutrient and herbivore exclusion experiments were executed at different locations to avoid crowding and interference between different experimental manipulations. At both sites, the coral *Montastraea annularis* Ellis and Solander, 1786 dominates the reef community and is frequently found bordering dense turf algae communities that are dominated by members of the orders *Gelidiaceae*, Gelidiellaceae, *Champiaceae*, *Lomentariaceae*, *Ceramiaceae* and *Bryopsidaceae*. Cyanobacteria were commonly present in the turf assemblages as well.

### Experimental approach

The rate of turf algal overgrowth of corals and the effect of turf algal presence on the health of neighbouring corals (measured as effective photochemical efficiency of polyps: see below) were measured for a 3–6 week period and the effects of herbivore exclusion and nutrient enrichment on this interaction were quantified. To obtain an island-wide estimate of turf algal abundance, the abundance of dominant benthic groups was quantified at 8 sites along the Leeward coast of Curaçao (Figure 1). At each site, 30 quadrats (0.5×1.0 m) were randomly positioned along a 75 m transect between depths of 7–10 m and photographed using a high-resolution digital camera. Photographs were analyzed using the program CPCe V3.6 to estimate the percentage cover of each benthic group (i.e. turf algae, macroalgae, coral, sand and other) using a point-intercept approach based on 50 randomly placed points in each photograph.

### Quantification of turf algal overgrowth and coral stress

The movement of the turf algae-coral boundary was measured using photographic time-series. Each week pictures were taken of a 15×10 cm area that included the turf algae-coral boundary and a ruler and two permanently installed nails for scale. By overlaying the sequence of pictures through time in Photoshop (ADOBE), the movement of the turf algae-coral boundary could be quantified (i.e., as speed in mm 3 wk^−1^ and as absolute direction toward/away from the coral). Interactions between corals and crustose coralline algae (CCA) were used as controls [14]. Measurements of turf-coral interactions were taken at weekly intervals around midday (11.00AM–13.00PM) from April 21st until June 1st 2009. Photographs of coral-CCA interactions were taken starting on May 11th 2009.

Pulse amplitude modulated [PAM] fluorometry was used to determine whether turf algae inflicted physiological stress on neighbouring corals (e.g., [38]–[40]). The effective photochemical efficiency (ΔF/Fm') i.e., the efficiency of Photosystem II of the endosymbiotic zooxanthellae in polyps under ambient light was used as a proxy for the “fitness” of the coral holobiont [41]–[43]. Effective photochemical efficiencies were measured *in situ* using a waterproof PAM fluorometer (Diving PAM, Walz GmbH). Measurements were taken on polyps within 1.0 cm of the coral-turf algal boundary at weekly intervals around midday (11.00AM–13.00PM) from April 21st until June 1^st^ 2009. Because CCA have little to no detrimental effects on neighboring corals [14], the effective photochemical efficiencies of coral polyps at similar positions on colonies bordering CCA were used as controls.

### Experiment 1: Nutrient enrichment

To determine if nutrient enrichment enhanced the overgrowth of coral by turf algae, we placed small packets (made out of nylon stockings) filled with Osmocote slow-releasing fertilizer (65gr, 14% N, 14% P_2_O_5_, 14% K_2_O) at <30 cm distances (up current) from the coral-turf algal boundaries (n = 40). A simple aquarium test kit (TetraTest) was used to confirm that nutrient release occurred for the duration of the experiment. Control packets (empty pieces of nylon stocking attached to the substrate with metal nails) were placed near similar coral-turf algae boundaries (n = 40). To distinguish between the effects of turf algae and nutrient enrichment on coral health, we included two additional treatments whereby corals bordering CCA were also subjected to a no nutrient and nutrient enriched treatment. CCAs were chosen because they are believed to have little to no negative effects on corals [14], [15]. In total, we followed 160 interactions through time following the methods described above.

### Experiment 2: Herbivore exclusion

Galvanized mesh was used to manufacture 40 herbivore-exclusion cages (30×30×15 cm [L×W×H], mesh size 0.5×0.5 cm). Half of these cages (n = 20) had no top panel allowing herbivorous fish to enter the cage, and served as controls (i.e., for the presence of metal). In the full cage treatment, mesoherbivores (i.e., fish >0.5 cm in height and/or width) could no longer access the coral-turf algal boundary. Preliminary experiments using time-lapse videography confirmed that herbivorous fish fed inside the open cages at rates similar to plots where cages were absent altogether (unpubl. data). Cages were randomly placed over turf-coral boundaries in a 50 by 50 m area between depths of 5–10 m. Cages were cleaned with a brush on a weekly basis during the first 3 weeks of the experiments and twice a week thereafter to reduce fouling. Cages did not significantly change water movement based on dissolution of clodcards [44]. Light levels inside the cage were similar to ambient light levels at the same depth as determined with the LI-COR quantum sensor of a diving PAM (Heinz Walz GmbH, Germany). The height of the turf algae was also measured with a ruler at weekly intervals between May 4th and June 3rd to determine if turf algae biomass increased when herbivores were excluded. Canopy height (in mm) was defined as the average height measured at three haphazardly selected positions within the turf algae canopy. To confirm that herbivorous fishes indeed preyed on turf algae, the feeding behaviour of the most abundant surgeonfish (*Acanthurus coeruleus* and *A. bahianus*) and parrotfish species (*Scarus vetula*, *S. taeniopterus*, and *Sparisoma viride*) was quantified *in situ*. Individual fish were followed for 5 min (n = 38) while noting the number of bites taken from either turf or macro algae.

### Statistics and analyses

Overgrowth rates were analyzed only at the end of the study due to the slow movement of interaction boundaries through time. Because interactions between corals and crustose coralline algae were only followed for 3 weeks, all overgrowth rates were expressed per 3 week intervals to allow straightforward comparisons. The effects of nutrient enrichment in combination with neighbouring algal type and herbivore exclusion on the movement of the interaction boundary between corals and neighbouring algae were analyzed using factorial ANOVAs on untransformed data. Differences in effective photochemical efficiency (ΔF/Fm') which was used as a proxy for coral “fitness” were analyzed using repeated measures (RM) ANOVA in both the nutrient enrichment and the herbivore exclusion experiment.

To determine whether five common herbivorous fishes preferred turf algae over macro algae, the grazing intensity (i.e., the number of bites taken from either algal group per 5 min) was compared using a factorial ANOVA where fish species and algal type were used as categorizing factors followed by post-hoc analyses (Tukey HSD).

No comparisons were made between the two sites because different experimental treatments were conducted at each site.

## Results

### Abundance of turf algae

**Figure 2:**
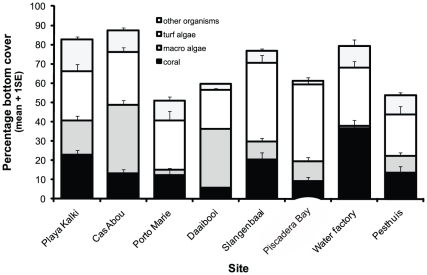
The abundance of turf algae relative to all other benthic groups along Curaçao's south-west shore. The abundance of five major functional groups was quantified between depths of 7 and 10 m (for an overview of the locations of surveyed sites, see Figure 1).

Turf algae were the most abundant living benthic group at five of eight surveyed sites along Curaçao's south-western shore (Figure 2), while macroalgae were most abundant at two sites (Cas Abou and Daaibooi) and coral at only one (Water Factory). Turf algal cover per site ranged between 20.3–41.0% and turf algae were the most abundant living benthic group (28.9%, SD: 7.8, n = 8) across all sites with 1.99 times higher cover than macro algae (14.6%, SD: 12.5, n = 8) and 1.73 times higher cover than corals (16.7%, SD: 9.7, n = 8). Only sand was a more abundant component of the reef bottom (31.1%, SD: 14.2, n = 8) between depths of 7–10 m.

### Experiment 1: Effects of nutrients on turf algal overgrowth

**Figure 3:**
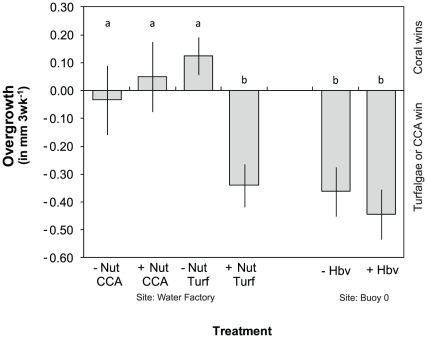
Overgrowth rates when turf algae are present (Turf) or absent, i.e., corals border crustose coralline algae (CCA). The treatment in which corals bordered CCA's served as “controls” for the turf algal treatment. The presence and absence of added nutrients is indicated by +Nut and -Nut respectively and the presence (+Hbv) or absence (-Hbv) of herbivores is indicated using the same methodology. Letters above the markers indicate significant groupings based on post-hoc analyses.

The superior competitor in each type of coral-algal interaction depended on the type of algae present and whether or not nutrients were provided (Figure 3). In coral-CCA interactions, overgrowth in either direction approached zero (range −0.03 − 0.05 mm 3 wk^−1^) and remained unchanged when nutrients were added (Table 1). In coral-turf interactions, corals overgrew turf algae at an average rate of 0.12 mm 3 wk^−1^ when no nutrients were added (Figure 3). When nutrients were added, the direction of competitive dominance reversed; turf algae became competitively superior and overgrew corals at an average rate of 0.34 mm 3 wk^−1^. Therefore, in the presence of increased nutrients, turf algae overgrew corals nearly three times faster than corals overgrew turf at control (ambient) nutrient concentrations. In sum, corals only became competitively inferior when extra nutrients and turf algae were present simultaneously (Table 1).

**Table 1:** Factorial ANOVA results of the effects of neighbour type (CCA or turfalgae) and nutrient enrichment on turfalgal overgrowth.

	df	MS	F	p
Neighbour (Ne)	1	0.61	1.31	ns (0.25)
Nutrients (Nu)	1	1.64	3.51	ns (0.06)
Ne × Nu	1	3.39	7.25	<0.01
Error	242	0.47		

ns  =  not significant.

In the absence of nutrient enrichment, turf algae did not overgrow neighboring corals, but the presence of turfs did lower the fitness of these corals (Table 2). The effective photochemical efficiency of corals bordering CCA was slightly, but significantly, higher (ΔF/Fm': 0.660, SE: 0.004; n = 80) compared to that of corals bordering turf algae (ΔF/Fm': 0.636, SE: 0.005; n = 76). When coral bordered CCA, the addition of nutrients lowered the average effective photochemical efficiency of neighboring corals (ΔF/Fm': 0.648, SE: 0.010; n = 79), but this difference was not significant (Table 2). This suggests that local nutrient enrichment alone does not cause the weakening of nearby corals but the combination of local nutrient enrichment and turf algae overgrowth does reduce coral fitness. When coral bordered turfalgae, the addition of nutrients also lowered the average effective photochemical efficiency of neighboring (ΔF/Fm': 0.634, SE: 0.007; n = 78), but this difference was also not significant (Table 2).

**Table 2:** Repeated-measures ANOVA results of the effects of neighbour type (CCA or turfalgae) and nutrient enrichment on the photosynthetic efficiency of corals.

	df	MS	F	p
Neighbour (Ne)	1	0.0458	5.47	<0.05
Nutrients (Nu)	1	0.0029	0.35	ns (0.55)
Ne × Nu	1	0.0021	0.25	ns (0.62)
Error	150	0.0084		

ns  =  not significant.

### Experiment 2: Effects of herbivore exclusion on turf algal overgrowth

**Figure 4:**
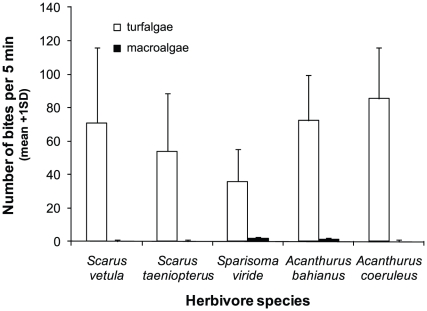
Food preference of five common herbivorous fishes: *Scarus vetula* (queen parrotfish), *Scarus taeniopterus* (princess parrotfish), *Sparisoma viride* (stoplight parrotfish), *Acanthurus bahianus* (ocean surgeonfish), *Acanthurus coeruleus* (blue tang).

At Buoy 0, the exclusion of herbivores did not influence the growth of turf algae (Table 3) but turf algae were the superior competitors in both control and herbivore exclusion treatments. The rate of turf algal overgrowth ranged between 0.36 and 0.44 mm 3 wk^−1^ which was higher than at the “Water Factory”, where corals overgrew turf algae in ambient conditions with herbivores present (Figure 3). Effective photochemical efficiencies did not differ (Table 4) between treatments where herbivores were present (ΔF/Fm': 0.535, SE: 0.011, n = 20) or excluded (ΔF/Fm': 0.557; SE: 0.009; n = 20). Turf algae obtained greater height in the herbivore exclusion treatment (Wilks' Lambda  = 0.70, F_5,34_ = 2.89; p<0.05). When herbivores were excluded, average canopy height averaged 9.9 mm (SE 1.2; n = 20) versus 5.9 mm (SE 0.6; n = 20) in grazed cages. Additional field observations confirmed that the five herbivorous fish considered in this study (*Scarus vetula*, *S. taeniopterus*, *Sparisoma viride*, *Acanthurus bahianus* and *A. coeruleus*) preferably grazed on turf algae rather than macro algae (Figure 4) and that grazing intensity on turf algae differed among fish species (Factorial ANOVA, species × algal type, F_4,68_ = 3.13, p<0.05). Both acanthurids and *Scarus vetula* showed higher grazing rates (mean bite rate 71–86 bites 5 min^−1^) relative to the other two species (mean bite rate 36–54 bites 5 min^−1^) based on post-hoc analyses (Tukey's HSD; p<0.05).

**Table 3:** One way ANOVA results of the effects of herbivore exclusion on turfalgal overgrowth.

	df	MS	F	p
Herbivore exclusion	1	0.420	1.912	ns (0.18)
Error	38	0.219		

ns  =  not significant.

**Table 4:** Repeated-measures ANOVA analysis of the effects of herbivore exclusion on the photosynthetic efficiency of corals.

	df	MS	F	p
Herbivore exclusion	1	0.019	2.073	ns (0.16)
Error	38	0.009		

ns  =  not significant.

## Discussion

Turf algae are the most dominant benthic group on Curaçaoan reefs and they are capable of overgrowing and reducing the fitness of the reef building coral *Montastraea annularis*. Only at one of two experimental sites (Water Factory) and in absence of experimental nutrient additions, were corals capable of overgrowing turf algae (Figure 3). However, turf algae were competitively superior under similar circumstances (i.e., no experimental nutrient additions and with herbivores present) at the other site (Buoy 0) suggesting that other spatially variable factors, other than the ones considered here, influence the outcome of coral-turf algal competition.

While visible overgrowth of corals by turf algae did not always occur, corals in close contact (i.e., <1 cm) with neighbouring turf algae showed lower effective photochemical efficiencies, which is commonly considered as a proxy for coral “fitness” [41]–[43]. An earlier study from Mexico on a sibling species of *Montastraea annularis*, (*M. faveolata*) also showed that turf algae had negative effects on neighbouring corals [27] by causing a decrease in the coral's zooxanthellae density, Chlorophyll *a* concentration, and tissue thickness. Healthy *M. faveolata* transplants were always overgrown by the turf algae, and in some cases killed [27]. Both these and our observations indicate that the important reef building species of the Caribbean genus *Montastraea* are directly stressed by neighbouring turf algae despite the fact that *Montastraea* species were historically characterized as competitively superior species [45]. Turf algae are theorized to negatively influence the growth, reproduction, and feeding efficiency of corals, which would explain the reduction in coral fitness observed in this study [23], [24], [46], [47].

Parrotfish (Scaridae), surgeonfish (Acanthuridae) and the long-spined sea urchin (*Diadema antillarum*) are the most abundant herbivores on Caribbean reefs, including those on Curaçao [12], [48], [49], and their ability to prevent the proliferation of macroalgae has been shown by many studies (e.g., [1], [27], [50]–[52]). The importance of eutrophication as a factor increasing the local abundance of macro algae is less clear and often disputed [16], [33], [34]. Here we showed that herbivores had no measurable impact on competitive overgrowth between corals and turf algae, but nutrient additions did. As such, turf algae and macro algae seem differently affected by factors generally believed to increase algal abundance, i.e., reduced herbivory and increased nutrient availability. Turf algae are more sensitive to the latter; whereas macroalgae respond more strongly to the former, though both factors likely drive the abundance of both algal groups to some extent.

Turf algal overgrowth progresses extremely slowly and therefore remains unnoticed unless studies are conducted over longer time periods and on small spatial scales. Based on our results, turf algal overgrowth progresses at rates between 1.8 and 2.2 cm per year, which is faster than the growth rate of most Caribbean corals, including the *Montastraea* species used in this study (0.9–1.2 cm yr^−1^; [53]–[55]). The competitive advantage of turf algae over reef building corals increases under eutrophied conditions.

Turf algae overgrew corals even in absence of added nutrients at Buoy 0. This site is located near a bay (Figure 1), in which nutrient conditions are generally higher than on the reef ([56] and references therein). When water exits the bay, the prevailing currents drive it directly to Buoy 0, causing nutrient concentrations to increase relative to background concentrations. Turf algal cover is known to respond significantly to fertilization elsewhere [16], [29], suggesting that turf algae can become competitively superior under eutrophied conditions that could arise semi-naturally (Buoy 0) or through experimental nutrient additions (Water Factory).

The presence of turf algae always reduced the fitness of neighbouring corals. It is important to note that turf algae impose stress even when “visible” overgrowth does not occur and this will go unnoticed in visual surveys of reef community structure. Coral growth rates observed in this study exceed the growth rates of some CCA species (1.5–2.5 cm yr^−1^), therefore coral growth is more likely to occur when corals border CCA than when they border turf algae. The neighbouring competitors surrounding a coral will therefore to some degree determine the coral's future abundance. Site specific averages of the abundances of benthic groups such as corals and algae do not provide information on their spatial configuration and thus the level of interaction between the groups. As such, they cannot be used to reliably predict the outcome of local competitive processes.

Because turf algae have become the most abundant benthic group on many reefs in the Caribbean ([12], [57]–[59], this study), the outlook for *Montastraea* species—which are the main reef building species in the region—is poor. In recent decades, turf algae have increased in abundance in locations around the world (e.g., Line Islands: [8], Brazil: [60], Hawaii: [4] and [16] with references therein), but their negative effects on corals may not always be obvious. On the Great Barrier Reef, McCook et al observed that turf algae had no negative effect on coral growth and corals were competitively superior under a wide range of environmental conditions [5]. We also observed that coral growth rates remain unchanged by neighbouring turf algae under some conditions (Figure 3), however, turf algae always lowered the “fitness” of neighbouring corals. Thus, the suite of negative effects exerted by turf algae on neighbouring includes these invisible effects, which must be considered in order to rightly conclude that turf algae do or do not have a negative effect on neighbouring corals. Furthermore, it is noteworthy that many of the studies showing corals' ability to overgrow turf algae are more than 30 yrs old ([5] and references therein) and were likely conducted in a time when most reefs had not experienced the level of degradation seen today. Increased nutrient availability increased the competitive ability of turf algae in our study (Figure 3) and the general eutrophication of reef waters could have additionally contributed to an environmental setting in which the competitive advantage of turf algae has increased relative to historic levels. Because the environment in which competitive interactions occur has changed, observations on competitive dynamics made in the past are potentially ill-suited to explain present day phenomena.

While the effects described in this study should not be generalized, we feel that the competitively superiority of this often neglected, but dominant functional benthic group (turf algae) deserves more attention in marine conservation efforts and studies on benthic dynamics of coral reef communities.

Here we showed for the first time that within our experimental context, turf algal overgrowth increases in response to nutrient enrichment and that herbivorous fish had no measurable effect on the rate of overgrowth, despite the fact that they preferred turf algae over macro algae. Because turf algae are abundant and often the most dominant benthic space occupiers of present day reef communities the ecological role of turf algae deserves more attention in studies on reef health and changes therein.
